# MALDI-MSI-Guided Laser Capture Microdissection Coupled with MS for Integrated Spatial Multi-Omics in Mouse Brain

**DOI:** 10.3390/life16071177

**Published:** 2026-07-16

**Authors:** Byoung-Kyu Cho, Jessica K. Lukowski, Minsoo Son, Antonia Zamacona Calderon, Moshe Levi, Katherine Stumpo, Savannah Snyder, Michael Esterling, Young Ah Goo

**Affiliations:** 1 Mass Spectrometry Technology Access Center at McDonnell Genome Institute, Washington University School of Medicine, St. Louis, MO 63108, USA; byoung-kyu@wustl.edu (B.-K.C.); lukowski@wustl.edu (J.K.L.); son.m@wustl.edu (M.S.); antonia@wustl.edu (A.Z.C.); 2 Department of Biochemistry and Molecular Biophysics, Washington University School of Medicine, St. Louis, MO 63108, USA; 3 Department of Biochemistry and Molecular and Cellular Biology, Georgetown University, Washington, DC 20057, USA; ml1742@georgetown.edu; 4 Meso Scale Diagnostics LLC, Rockville, MD 20850, USA; kstumpo@gmail.com; 5 Bruker Daltonics, Billerica, MA 01821, USA; savannah.snyder@bruker.com (S.S.); michael.easterling@bruker.com (M.E.); 6 Department of Genetics, Washington University School of Medicine, St. Louis, MO 63108, USA

**Keywords:** spatial multi-omics, matrix-assisted laser desorption/ionization, mass spectrometry imaging, laser capture microdissection, LC-MS/MS

## Abstract

Spatial multi-omics analyzes biomolecules such as the proteome, metabolome, and lipidome within their native spatial context in tissues or cells. Mass spectrometry imaging (MSI) has emerged as a powerful technique for mapping the region-specific molecular distribution in regions of interest (ROIs). Laser capture microdissection coupled with mass spectrometry (LCM-MS) is another well-established workflow, enabling the accurate characterization of biomolecules in ROIs. To advance the current analytical application, we expanded a matrix-assisted laser desorption/ionization (MALDI)-MSI-guided LCM-MS workflow for integrated multi-omics analysis and applied it to mouse brain tissue as a proof-of-principle validation. MALDI-MSI annotated 387 putative metabolites and lipids, revealing distinct molecular distributions between the cortex and hippocampus. Both regions were subsequently isolated as ROIs using LCM and analyzed by LC-MS/MS metabolomics, lipidomics, and proteomics to achieve accurate biomolecular profiling. LC-MS/MS metabolomics and lipidomics annotated 249 compounds, several of which exhibited distinct abundance patterns between the two regions. LC-MS/MS proteomics matched to over 3500 protein groups across the two regions. Biological network analysis revealed strong associations between molecular pathways and known region-specific phenotypes. Overall, this MALDI-MSI-guided LCM-MS workflow enables comprehensive spatial multi-omics profiling and quantitative biomolecular analysis, providing valuable insights into complex biological systems and spatial molecular organization.

## 1. Introduction

Spatial multi-omics has revolutionized biomolecular analysis by preserving molecular spatial context within a given biospecimen such as tissue or cell [1,2]. It aims to elucidate the roles of metabolites, lipids, and proteins, and their interplay within regions of interest (ROIs) in the specific biological microenvironment [3,4,5]. Therefore, this approach provides valuable insights and generates hypotheses for a better understanding of biological networks through a comprehensive perspective, as biomolecules maintain their native state and placement.

Mass spectrometry imaging (MSI) has advanced significantly in the spatial multi-omics field to map the molecular distribution without labeled probes or prior knowledge of the target molecules [6,7,8]. It has expanded to a variety of biological research areas, including neuroscience [9,10], cancer biology [1,11,12], and drug discovery [13,14]. In particular, MALDI-MSI is an advanced analytical technique with its high spatial resolution and a broad *m/z* range, allowing for the analysis of small metabolites, lipids, peptides, and intact proteins with a spatial resolution down to 5 µm [15,16]. Despite these strengths, MALDI-MSI has limitations in untargeted omics applications. MALDI-MSI relies on MS1 acquisition, which introduces the ambiguity of biomolecular annotation [17]. While MS/MS acquisition is applicable to MALDI-MSI, it is a time-intensive process, making it challenging to achieve high spatial resolution. Furthermore, advancements in proteomics are particularly essential, as sample preparation remains challenging, and the sensitivity of MALDI-MSI lags compared to traditional LC-MS/MS-based proteomics.

Laser capture microdissection coupled with mass spectrometry (LCM-MS) is another powerful approach utilized in spatial multi-omics. LCM enables the precise isolation of specific ROIs, where biomolecules are extracted and subjected to LC-MS/MS analysis to profile metabolites, lipids, and proteins while preserving tissue architecture [18,19,20,21,22]. While MALDI-MSI offers visual spatial precision, LCM-MS has distinct advantages over it. A key strength of LCM-MS is its reliance on MS/MS acquisition, enabling accurate qualitative and quantitative analysis of biomolecules. In addition, LCM-MS has become an exceptionally sensitive technique, particularly beneficial in proteomics, with advancements in the latest MS technology [18,23]. Thousands of proteins, including low-abundance ones that might be missed by MALDI-MSI, can be identified using the LCM-proteomics approach.

**Figure 1:**
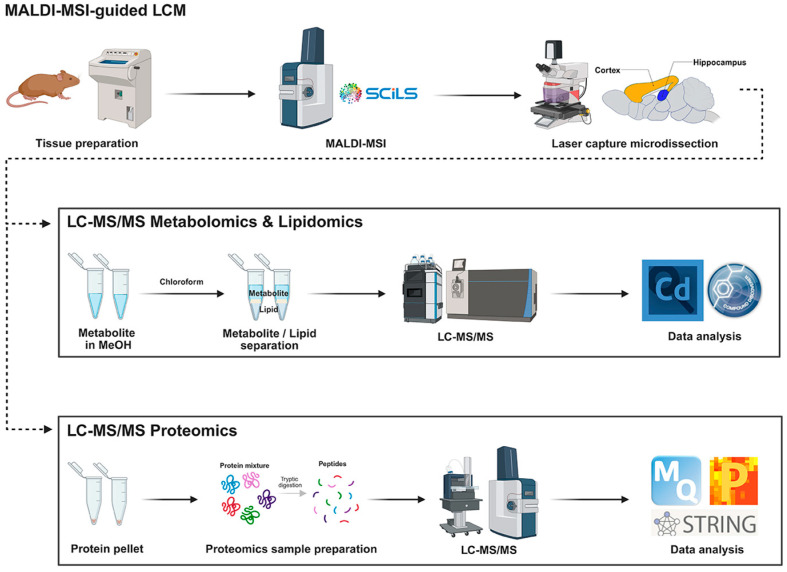
Overall workflow of MALDI-MSI-guided LCM-MS. Mouse brain tissue sections were analyzed using MALDI-MSI, identifying two distinct spatial regions, the cortex and hippocampus. These regions were isolated by LCM in methanol. The aqueous phase of the LCM samples was further separated into metabolite and lipid fractions, which were analyzed by LC-MS/MS for metabolomics and lipidomics, respectively. The protein pellet from the same LCM samples was trypsin-digested and analyzed by LC-MS/MS for proteomics.

To advance the current spatial multi-omics applications, we present a MALDI-MSI-guided LCM-MS approach. As a proof of concept, we applied it to mouse brain tissue to evaluate its ability to profile region-specific biomolecules. The overall scheme is illustrated in Figure 1. Briefly, two ROIs, the hippocampus and cortex, were determined based on the spatially distinct distribution of metabolites and lipids through MALDI-MSI and confirmed through H&E staining. These ROIs were isolated using LCM, and metabolites, lipids, and proteins were extracted from the same LCM-collected sample. Each type of biomolecule was subsequently analyzed by LC-MS/MS using its own optimized platform. Through this approach, we aimed to demonstrate the capability of our spatial multi-omics approach to provide region-specific characterization of metabolites, lipids, and proteins.

## 2. Materials and Methods

### 2.1. Materials

The six-month-old male C57BL/6J mouse was purchased from The Jackson Laboratory (Bar Harbor, ME, USA). Indium tin oxide (ITO)-coated glass slides were purchased from Bruker Daltonics (Billerica, MA, USA), and polyethylene naphthalate (PEN) membrane slides were purchased from Leica Microsystems (Deerfield, IL, USA). Dithiothreitol (DTT) and trypsin were purchased from Promega (Madison, WI, USA). Iodoacetamide (IAA) was purchased from Millipore Sigma (St. Louis, MO, USA). Acetonitrile (ACN), C18 spin columns, chloroform, dihydroxybenzoic acid (DHB), formic acid (FA), methanol (MeOH), N-(1-naphthyl) ethylenediamine dihydrochloride, urea, and water were all purchased from Thermo Scientific (Allentown, PA, USA).

### 2.2. MALDI-MSI

A fresh frozen mouse brain tissue was mounted on a cryomicrotome chuck by freezing a small droplet of water and then sectioned using a blade and a specimen temperature of −18 °C. Tissue was sectioned (Cryostar NX70, Leica, Deerfield, IL, USA) at a 10 μm thickness and placed on an ITO-coated glass slide for MALDI-MSI. Three consecutive serial sections were prepared from the same anatomical level. The first and second serial sections were mounted on indium tin oxide (ITO)-coated glass slides for positive- and negative-ion MALDI-MSI, respectively, and the third serial section was mounted on a PEN membrane slide for subsequent laser capture microdissection (LCM). The PEN membrane slide was stored at −80 °C until analysis. ITO-coated glass slides were placed in a desiccator for 30 min before matrix application. A M3 TM-Sprayer (HTX Technologies, Chapel Hill, NC, USA) was used for MALDI matrix application. For positive-ion MALDI-MSI, DHB (15 mg/mL in 90% ACN with 0.1% TFA) was used. Spraying conditions were as follows: 60 °C nozzle temperature, flow rate of 0.09 mL/min, 12 passes, a N_2_ pressure of 10 psi, track spacing of 3 mm and a 40 mm distance between the nozzle and sample was maintained. For negative-ion MALDI-MSI, the adjacent serial section was coated with N-(1-naphthyl) ethylenediamine dihydrochloride (10 mg/mL in 70% methanol). Spraying conditions were as follows: 80 °C nozzle temperature, a flow rate of 0.095 mL/min, 12 passes, a N_2_ pressure of 10 psi, a track spacing of 3 mm and a 40 mm distance between the nozzle and sample was maintained. MALDI-MSI was performed on a timsTOF Flex MALDI2 (Bruker Daltonics) equipped with a SmartBeam 3D laser source in positive and negative ion mode using 200 shots/pixel, 60% laser power, and 20 μm pitch between pixels. The instrument was externally calibrated using ESI Low Concentration Tuning Mix and operated with a mass range of 50–1500 *m*/*z*. MALDI-MSI data was imported into the SCiLS software (Bruker Daltonics, Version 2025b) and converted into the imzML format with spectra restriction using only the m/z intervals of the imported peaks. The resulting imzML and ibd files were analyzed against SwissLipids, HMDB, CoreMetabolome, and LipidMaps databases in METASPACE. Unsupervised segmentation was performed in SCiLS to visualize molecular distinctions between anatomical regions and to support the identification of regions of interest for subsequent analyses.

### 2.3. Laser Capture Microdissection

Prior to LCM, the sections from the MALDI-MSI analysis were stained with hematoxylin and eosin (H&E) to confirm ROIs for LCM isolation. Briefly, the section was washed in 1× TBS, 70% EtOH, and distilled water before being placed in hematoxylin for 45 s. The section was then rinsed with distilled water and 1× TBS, immersed in 70% and 95% ethanol, counterstained with eosin (1:2 dilution in ethanol), dehydrated in 95% and 100% ethanol, cleared in xylene, and cover-slipped. H&E-stained slides were then scanned at 20× on an Olympus BX 61 vs. Scanner. The PEN membrane slide was placed in a desiccator for 30 min prior to LCM. Traditionally, slides are washed with a gradient of ethanol and xylene prior to LCM isolation, but those steps were omitted due to our LC-MS/MS metabolomics workflow. LCM was performed on a Leica LMD7000 microscope with the following cutting parameters: power at 60, aperture set between 17 and 20, speed ranging from 8 to 12, bridge size set to 0, a final pulse at 6, head current at 100%, and pulse frequency between 120 and 150. Approximately 1.3 × 10^6^ μm^2^ of tissue was collected from each of the cortex and hippocampus into 40 μL of 80% methanol from one tissue section.

### 2.4. LC-MS/MS Metabolomics and Lipidomics

The resulting LCM sample was homogenized using a pulsed tip sonicator on dry ice. The protein pellet formed was saved for LC-MS/MS proteomics. The protein-free supernatant was transferred to 2 mL glass tubes and dried in a vacuum concentrator without heating. A pre-chilled MeOH:chloroform solution (1:2, *v*/*v*) was added, followed by vigorous vortexing and centrifugation at 2900× *g* for 10 min at 4 °C. The bottom chloroform layer, containing lipids, was transferred to new 2 mL glass tubes and dried in a vacuum concentrator without heat. The upper methanol layer, containing metabolites, was dried under the same conditions. Three types of analytical columns were used for LC-MS/MS metabolomics and lipidomics analysis. For metabolite analysis, two columns were utilized: the Waters™ Acquity HSS T3 C18 column (2.1 mm × 150 mm, 1.8 μm) for hydrophobic metabolites and the Waters™ Acquity BEH Amide VanGuard FIT column (2.1 mm × 150 mm, 1.7 μm) for hydrophilic metabolites. For the C18 column, mobile phase A was 0.1% formic acid (FA) in water, and mobile phase B was 0.1% FA in ACN. Samples were separated with a 9-min analytical gradient ranging from 2% to 27% of mobile phase B at a flow rate of 500 μL/min. For the amide column, mobile phase A consisted of 20 mM ammonium acetate in water (pH 9), and mobile phase B was ACN (pH 9). Samples were separated with a 10-min gradient ranging from 95% to 80% of mobile phase B at a flow rate of 400 μL/min. For lipid analysis, a Waters™ BEH C8 column (2.1 mm × 100 mm, 1.7 μm) was utilized. Mobile phase A was composed of 10 mM ammonium acetate with 5% methanol and 0.1% acetic acid in water, while mobile phase B consisted of 0.1% acetic acid in methanol. Lipid samples were separated using a 10-min gradient ranging from 20% to 100% of mobile phase B at a flow rate of 400 μL/min. All MS/MS analyses were performed using an Orbitrap ID-X Tribrid Mass Spectrometer (Thermo Scientific) with AcquireX DeepScan, acquiring data in both negative and positive ion modes. Raw LC–MS data was processed using Compound Discoverer 3.3 (Thermo Fisher Scientific) for feature detection, retention time alignment, peak grouping, area calculation, elemental composition prediction, and compound annotation. Metabolite and lipid annotations were assigned based on accurate precursor mass (5 ppm), isotope pattern (>90%), elemental composition prediction match, and, when available, MS/MS spectral library matching against mzCloud (https://www.mzcloud.org/) (accessed on 13 July 2026), MassBank of North America (http://mona.fiehnlab.ucdavis.edu) (accessed on 13 July 2026), Global Natural Product Social Molecular Networking [24], and National Institute of Standards and Technology (NIST20) databases (cosine match factor > 0.5). Annotations supported by MS/MS spectral library matching were reported as Metabolomics Standards Initiative Level 2 putative annotations [25]. Features annotated only by accurate mass, predicted elemental composition, or database matching without MS/MS support were considered lower-confidence putative annotations and were not used for annotation.

### 2.5. LC-MS/MS Proteomics

The resulting pellet after the metabolite and lipid extraction from the same LCM sample was resuspended in 8M urea, reduced with DTT, and cysteines were alkylated with iodoacetamide (IAA). The sample was then diluted to <2M urea and digested with trypsin at 37 °C overnight. The resulting peptides were desalted using solid-phase extraction on a C18 spin column and eluted in 80% acetonitrile in 0.1% formic acid. Peptides were analyzed by LC-MS/MS using a nanoElute coupled with a timsTOF Pro2 Mass Spectrometer (Bruker Daltonics). Samples were loaded on a capillary C18 column (IonOpticks, Collingwood, Australia, 75 μm × 250 mm, 1.7 μm). The flow rate was kept at 300 nL/min. Mobile phase A was 0.1% FA in water, and mobile phase B was 0.1% FA in ACN. The peptides were separated on a 115-min analytical gradient from 2 to 35% of mobile phase B. The timsTOF Pro2 was operated in the PASEF mode. MS and MS/MS spectra were acquired from 100 to 1700 *m*/*z* using 10 PASEF MS/MS scans per cycle with a near 100% duty cycle. The inverse reduced ion mobility 1/K_0_ was set to 0.60–1.60 V·s/cm^2^ over a ramp time of 100 ms. The resulting tandem MS data was queried for protein identification and label-free quantification against the Swiss-Prot *Mus Musculus* database using MaxQuant (version 2.6.6.0) [26,27]. The following search parameters were applied: trypsin digestion with cleavage after K or R (except when followed by P), allowance for up to 2 missed cleavages, carbamidomethylation of cysteine (static modification), and variable modifications including oxidized methionine, deamidated asparagine/glutamine, and N-terminal acetylation. Protein abundance values were obtained using Perseus software (version 2.0.7.0). LC–MS/MS analyses were performed in technical duplicate to assess analytical reproducibility. Because biological replicates were not available, no inferential statistical analyses were performed. Instead, differences in protein abundance between the cortex and hippocampus were evaluated descriptively using log_2_ fold change. Proteins with a log_2_ fold change >1 (higher abundance in the cortex) or <−1 (higher abundance in the hippocampus) were selected for downstream functional enrichment analysis. Functional enrichment analysis was performed using the Search Tool for the Retrieval of Interacting Genes/Proteins (STRING) database (version 12.0). The mass spectrometry proteomics data have been deposited in the ProteomeXchange Consortium via the PRIDE [28] partner repository with the dataset identifier PXD060073 and 10.6019/PXD060073.

## 3. Results

### 3.1. Region-Specific Molecular Distributions Matched by MALDI-MSI

**Figure 2:**
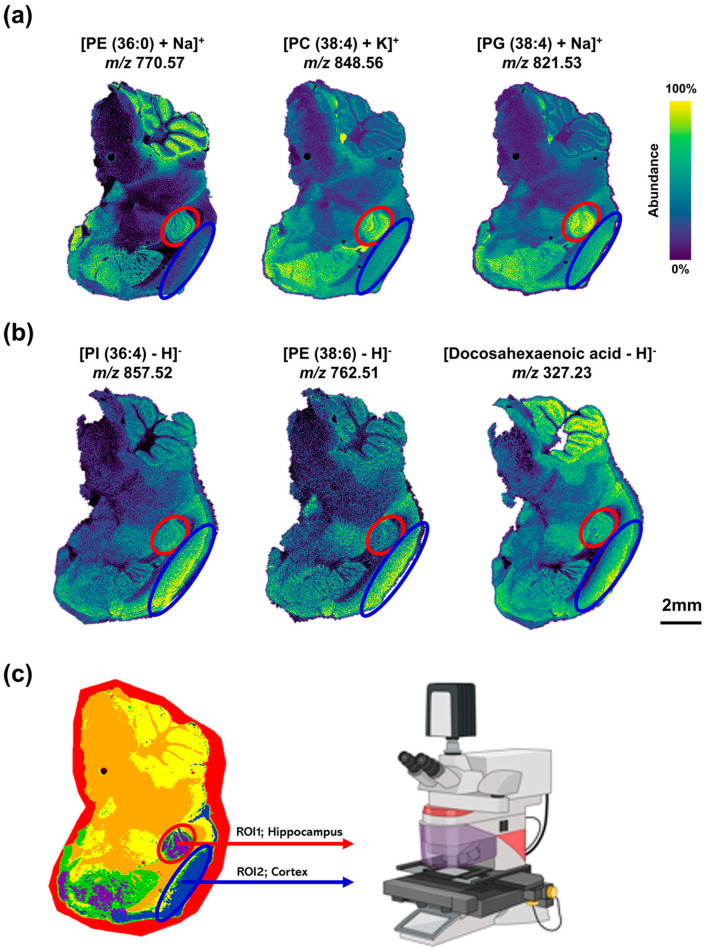
The distinct spatial distribution in mouse brain tissue was visualized by MALDI-MSI. (**a**) In the hippocampus, higher abundances of PE36:0, PC38:4, and PG38:4 were observed. (**b**) In the cortex, PI36:4, PE38:6, and docosahexaenoic acid were highly enriched. (**c**) Unsupervised spatial segmentation in SCiLS revealed molecularly distinct hippocampus and cortex regions, determined as ROIs for LCM. The hippocampus region is outlined in red, and the cortex region is outlined in blue.

To determine ROIs based on spatial molecular distribution, we initially performed MALDI-MSI on sectioned mouse brain tissue using both positive and negative ion modes at a spatial resolution of 20 μm. As a result, 203 and 184 putative metabolites and lipids were annotated at 20% FDR in METASPACE (Supplemental Table S1). Several compounds exhibited a distinct spatial distribution, especially in the hippocampus and cortex regions. For example, PE (36:0), PC (38:4), and PG (38:4) were highly expressed in the hippocampus (Figure 2a), whereas PI (36:4), PE (38:6), and docosahexaenoic acid showed a higher abundance in the cortex region (Figure 2b). In addition, unsupervised spatial segmentation performed in SCiLS highlighted the molecular distinctions between these anatomical regions (Figure 2c). While MALDI-MSI provides spatial visualization based on biomolecular distinctions, this data alone cannot provide confident biomolecular profiling as it is primarily an MS1-level technique. To achieve accurate biomolecule annotation, we selected both ROIs for LCM, followed by a traditional LC-MS/MS approach, including metabolomics, lipidomics, and proteomics. These anatomical regions were readily identifiable in brightfield images and were confirmed by H&E staining (Supplemental Figure S1). Subsequently, approximately 1.3 million µm^2^ of tissue was isolated from each hippocampus and cortex region using LCM for comparative molecular profiling.

### 3.2. Spatial Metabolomics and Lipidomics via LCM-LC-MS/MS

**Figure 3:**
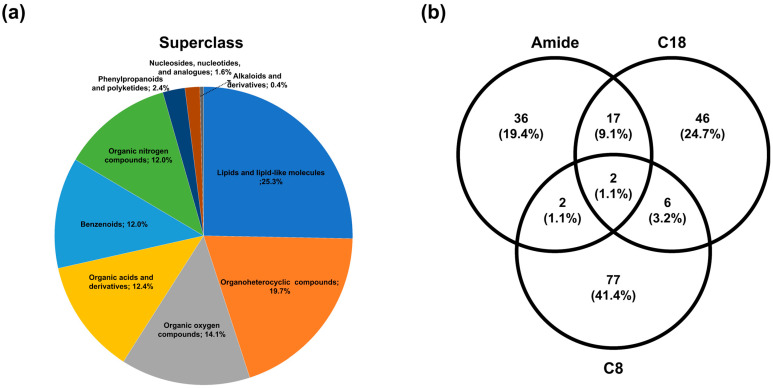
Annotated compounds from LCM metabolomics and lipidomics. (**a**) Superclass classification of annotated metabolites and lipids. (**b**) Venn diagram showing the distribution of annotated compounds across the three analytical column systems (C8, C18, and amide).

Metabolites were extracted using MeOH from the LCM-collected samples, and proteins were pelleted during the process. The metabolite contents were further separated using a mixture of MeOH and chloroform to isolate the organic phase, primarily containing lipids. Our integrated sample preparation workflow enables LC-MS/MS metabolomics, lipidomics, and proteomics from the same sample origin, minimizing biological variation for an unbiased multi-omics approach. We employed LC-MS/MS metabolomics and lipidomics with three analytical columns: C18 and amide columns for the aqueous phase and a C8 column for the organic phase. In total, 249 metabolites and lipids were annotated against mzCloud, GNPS, MoNA, and NIST20 using Compound Discoverer software 3.3. The classification of annotated molecules by superclass is shown in Figure 3a, and the full list of annotated compounds and their MS peak areas in each ROI is described in Supplementary Table S2. Specifically, 101, 82, and 66 compounds were matched from the C8, C18, and amide columns, respectively. Notably, only 27 compounds were commonly detected across two or three columns, while the majority of compounds, 159 in total, were uniquely matched within a specific column system when isomers were excluded (Figure 3b). Our data highlights the advantage of multiple analytical column systems to achieve comprehensive metabolite/lipid annotation. Comparison of the MS peak areas of annotated compounds between the cortex and hippocampus revealed differences in metabolite abundance. In particular, acetylcholine (ACh), acetyl-L-carnitine, inosine, and nicotinamide exhibited log_2_ fold changes greater than 2 in the hippocampus relative to the cortex. These metabolites have previously been implicated in hippocampal functions, including neuroprotection and the regulation of synaptic plasticity [29,30,31,32,33]. Because these observations were obtained without biological replicates, they should be considered preliminary and interpreted with caution. Future studies incorporating appropriate biological replication will be required to validate these observed abundance differences. Nevertheless, these findings demonstrate the potential of the LCM–LC–MS/MS workflow for spatial metabolomics and lipidomics.

### 3.3. Spatial Proteomics via LCM-LC-MS/MS

**Figure 4:**
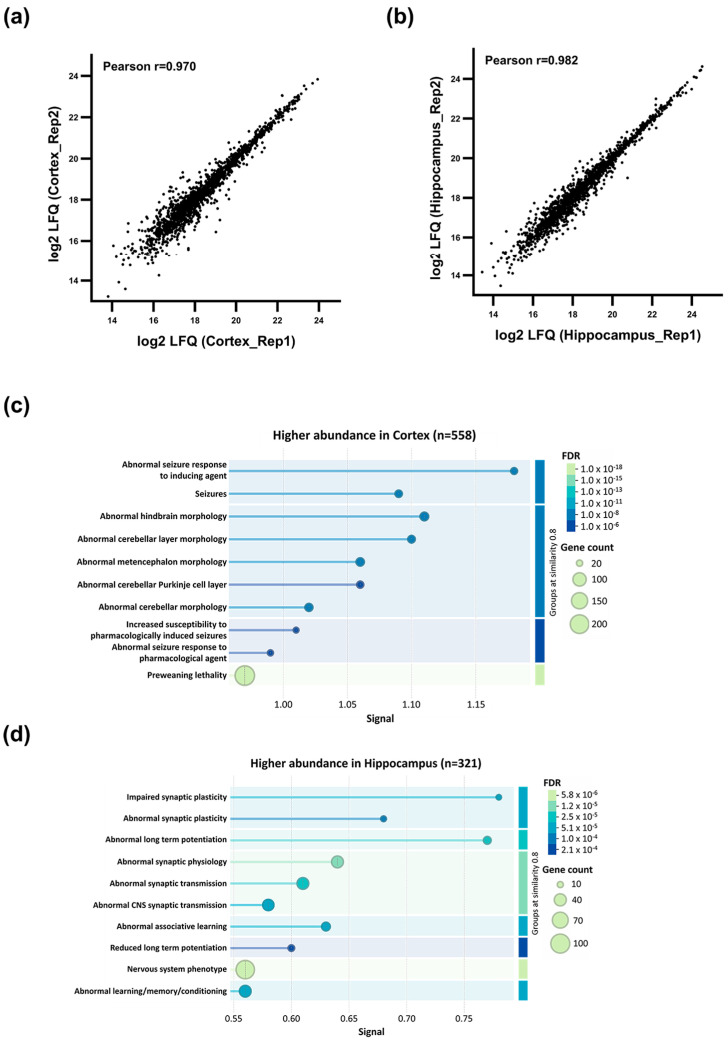
Technical reproducibility and Mammalian Phenotype Ontology analysis of region-enriched proteins matched by LCM–LC–MS/MS proteomics. Correlation plots of protein intensities between two technical LC–MS/MS replicates from the (**a**) hippocampus and (**b**) cortex, demonstrating analytical reproducibility. Top 10 enriched Mammalian Phenotype Ontology terms for proteins exhibiting higher abundance in the (**c**) hippocampus and (**d**) cortex. The top 10 enriched phenotype terms are shown based on the STRING enrichment analysis.

The protein pellet generated from the metabolite extraction was utilized for LC-MS/MS proteomics. Our LCM-based sample preparation workflow allows an unbiased multi-omics approach by using the same sample origin for the analysis of metabolomics, lipidomics, and proteomics. A total of 3522 protein groups were matched at a 1% false discovery rate (FDR) at both the protein and peptide-spectrum match (PSM) levels (Supplemental Table S3). LC–MS/MS analyses were performed in technical duplicate to assess analytical reproducibility. Pearson correlation coefficients between the technical replicates were 0.970 for the cortex and 0.982 for the hippocampus (Figure 4a,b), demonstrating high reproducibility of the proteomics workflow. Because biological replicates were not available, protein abundance differences between the cortex and hippocampus were evaluated descriptively using log_2_ fold change rather than inferential statistical testing. Based on a log_2_ fold change threshold of >1 or <−1, 558 proteins exhibited higher abundance in the cortex, whereas 321 proteins exhibited higher abundance in the hippocampus. These region-enriched protein sets were subsequently used for Mammalian Phenotype Ontology enrichment analysis. The Mammalian Phenotype Ontology analysis revealed distinct functional characteristics for each brain region (Figure 4c,d). Proteins with higher abundance in the cortex were enriched for phenotypes related to seizures and motor function, consistent with the established roles of the cerebral cortex in sensory processing, motor control, and higher-order brain functions [34,35,36]. In the hippocampus, proteins with higher abundance were enriched for phenotypes associated with long-term potentiation (LTP) and synaptic plasticity, consistent with the known physiological functions of the hippocampus and in agreement with metabolomics and lipidomics observations. Because these analyses were performed without biological replicates, the observed protein abundance differences and associated functional enrichments should be considered exploratory and interpreted with appropriate caution.

## 4. Discussion

MALDI-MSI provided region-specific molecular distributions within the mouse brain, particularly distinguishing the hippocampus and cortex based on lipid and metabolite localization patterns. While approximately 400 putative small molecules were matched from MALDI-MSI, confident molecular identification is inherently limited because it primarily relies on MS1 information. To address this limitation, MALDI-MSI was integrated with LCM followed by LC–MS/MS, enabling confident metabolite, lipid, and protein annotation from anatomically defined brain regions. Furthermore, the integrated sample preparation workflow allowed metabolomics, lipidomics, and proteomics analyses from the same sample origin, thereby minimizing biological variation and enabling unbiased multi-omics interpretation.

Although each omics dataset was analyzed independently, the results converged on consistent region-specific biological characteristics. Metabolomics revealed higher relative abundances of acetylcholine, acetyl-L-carnitine, inosine, and nicotinamide in the hippocampus compared with the cortex. These metabolites have previously been associated with neuronal energy metabolism, neuroprotection, neurotransmission, and the regulation of synaptic plasticity [29,30,31,32,33]. Consistent with these observations, proteomic analysis showed that proteins with higher abundance in the hippocampus were enriched for Mammalian Phenotype Ontology terms related to long-term potentiation (LTP) and synaptic plasticity. Although these datasets do not establish direct molecular interactions, their concordance suggests that independent metabolomic and proteomic measurements captured complementary aspects of hippocampal biology.

A similar agreement was observed for the cortex. While the metabolomic and lipidomic profiles demonstrated distinct molecular compositions relative to the hippocampus, proteins with higher abundance in the cortex were associated with Mammalian Phenotype Ontology terms related to seizures and motor function. These findings are consistent with the established physiological roles of the cerebral cortex in sensory integration, motor control, and higher-order cognitive processing [34,35,36]. Together, the metabolomic, lipidomic, and proteomic datasets independently matched molecular features that correspond to the known functional specialization of each brain region.

Among the hippocampal proteins, Gria1 and Gria2, which encode AMPA receptor subunits involved in excitatory synaptic transmission and LTP, exhibited higher abundance in the hippocampus [37,38]. Previous studies have demonstrated that cholinergic signaling modulates AMPA receptor trafficking and synaptic efficacy during hippocampal plasticity. Thus, the concurrent observation of increased acetylcholine abundance together with enrichment of synaptic plasticity-related proteins provides complementary molecular evidence supporting the established role of the hippocampus in learning and memory. While these observations should not be interpreted as evidence of direct causal relationships, they illustrate how integrating multiple molecular layers can provide a more comprehensive view of region-specific brain function.

Several limitations should be acknowledged. The metabolomics and lipidomics analyses were performed without biological replicates, and the proteomics analyses included only technical duplicates. Consequently, the observed abundance differences and functional enrichments should be regarded as exploratory rather than statistically validated biological differences. Future studies incorporating biological replication and quantitative cross-omics integration methods, such as pathway-level integration or network-based analyses, will be important for validating these findings and further elucidating the molecular interactions underlying regional brain function. Overall, this study demonstrates that integrating MALDI-MSI with LCM and LC–MS/MS enables complementary spatial metabolomic, lipidomic, and proteomic characterization from the same tissue source. Despite the exploratory nature of the current dataset, the concordant biological signatures observed across multiple omics layers highlight the potential of this workflow for investigating region-specific molecular organization in the brain.

## 5. Conclusions

In this study, we established a MALDI-MSI-guided LCM-LC-MS/MS workflow and demonstrated its application to spatial multi-omics profiling of the mouse brain. This approach enabled complementary metabolomic, lipidomic, and proteomic characterization of anatomically defined cortex and hippocampus regions from the same tissue source. The multi-omics datasets revealed region-associated molecular differences that were consistent with known biological functions of these brain regions, illustrating the potential of integrated spatial molecular profiling to provide complementary insights across multiple omics layers. While biological replicates under the specific biological perturbation system are needed to fully validate the robustness of the cross-omics integration, our proof-of-concept study highlights the potential of this approach for application to various biological systems, including neurodegenerative disorders and cancers. Overall, this integrated platform represents a promising strategy for spatially resolved molecular characterization and for advancing multi-omics investigations in biomedical research.

## Data Availability

All data is available upon request. The mass spectrometry proteomics data have been deposited to the ProteomeXchange Consortium via the PRIDE partner repository with the dataset identifier PXD060073 and 10.6019/PXD060073. This work was performed by the Mass Spectrometry Technology Access Center at the McDonnell Genome Institute (MTAC@MGI) at Washington University School of Medicine.

## Supplementary Materials

The following supporting information can be downloaded at: https://www.mdpi.com/article/10.3390/life16071177/s1, Figure S1: H&E-stained images confirming anatomical regions selected for laser capture microdissection, Table S1: Annotated list of 387 putative metabolites and lipids identified in mouse brain tissue using MALDI-MSI, Table S2: A list of 249 compounds annotated in the hippocampus and cortex regions of mouse brain tissue using LCM-LC-MS/MS-based metabolomics and lipidomics, Table S3: Identified list of 3522 protein groups in the hippocampus and cortex regions of mouse brain tissue using LCM-LC-MS/MS-based proteomics.
