# Supplementary figures and images for: MALDI-MSI-Guided Laser Capture Microdissection Coupled with MS for Integrated Spatial Multi-Omics in Mouse Brain
Source: Life (Basel). 2026 Jul 16;16(7):1177. doi: 10.3390/life16071177 (PMC13412975; doi:10.3390/life16071177)

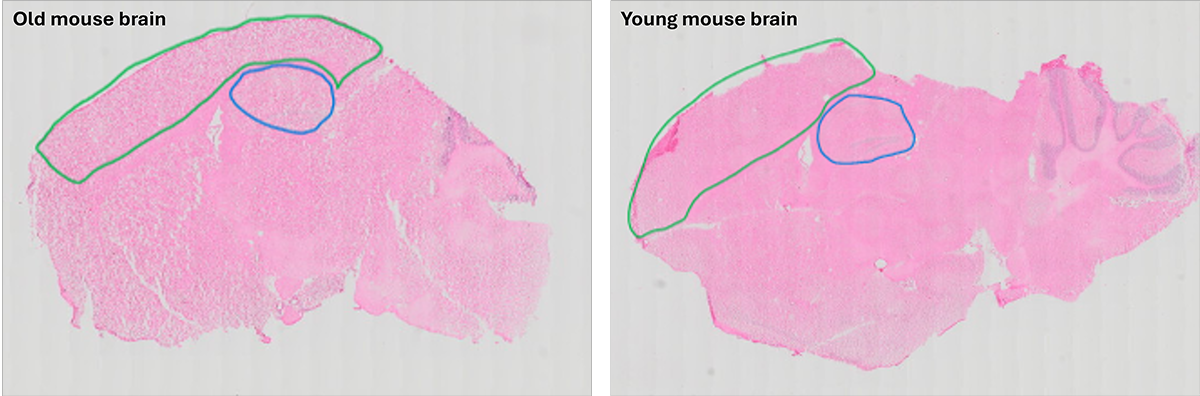

Supplement: Supplementary file 1 [file life-16-01177-s001.zip › Supplementary Figure S1.tif]
